# Cardiac injury is associated with inflammation in geriatric COVID‐19 patients

**DOI:** 10.1002/jcla.23654

**Published:** 2020-11-18

**Authors:** Xu Yan, Shuang Wang, Piyong Ma, Bo Yang, Daoyuan Si, Guohui Liu, Long Liu, Mei Ding, Wen Yang, Jiayu Li, Huan Sun, Ping Yang

**Affiliations:** ^1^ Cardiology Department China‐Japan Union Hospital of Jilin University Changchun China; ^2^ Jilin Provincial Molecular Biology Research Center for Precision Medicine of Major Cardiovascular Disease Changchun China; ^3^ COVID Medical Team of China‐Japan Union Hospital of Jilin University Changchun China; ^4^ Intensive care unit China‐Japan Union Hospital of Jilin University Changchun China; ^5^ Institute of Organ Transplantation Tongji Hospital Tongji Medical College Huazhong University of Science and Technology Wuhan China

**Keywords:** cardiac injury, COVID‐19, elderly patient, inflammation, troponin

## Abstract

**Background:**

Geriatric patients with coronavirus disease (COVID‐19) are at high risk of developing cardiac injury. Identifying the factors that affect high‐sensitivity cardiac troponin I may indicate the cause of cardiac injury in elderly patients, and this could hopefully assist in protecting heart function in this patient population.

**Methods:**

One hundred and eighty inpatients who were admitted for COVID‐19 were screened. Patients older than 60 years were included in this study, and the clinical characteristics and laboratory results of the cohort were analyzed. The correlation between cardiac injury and clinical/laboratory variables was statistically analyzed, and further logistic regression was performed to determine how these variables influence cardiac injury in geriatric patients.

**Results:**

Age (*p* < 0.001) significantly correlated with cardiac injury, whereas sex (*p* = 0.372) and coexisting diseases did not. Rising procalcitonin (*p* = 0.001), interleukin‐2 receptor (*p* < 0.001), interleukin 6 (*p* = 0.001), interleukin 10 (*p* < 0.001), tumor necrosis factor α (*p* = 0.001), high‐sensitivity C‐reactive protein (*p* = 0.001), D‐dimer (*p* < 0.001), white blood cells (*p* < 0.001), neutrophils (*p* = 0.001), declining lymphocytes (*p* < 0.001), and natural killer cells (*p* = 0.005) were associated with cardiac injury and showed predictive ability in the multivariate logistic regression.

**Conclusion:**

Our results suggest that age and inflammatory factors influence cardiac injury in elderly patients. Interfering with inflammation in this patient population may potentially confer cardiac protection.

## Graphical abstract

Our study explored potential risk factors related to cardiac injury in elderly patients with COVID‐19. The results suggest that age and inflammatory factors influence cardiac injury in elderly patients. Interfering with inflammation in this patient population may potentially confer cardiac protection.

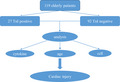

## 1 INTRODUCTION

Since the initial outbreak of the novel coronavirus disease (COVID‐19) in December 2019, the pandemic has emerged as an unprecedented global healthcare crisis, with a total of 45,428,731 cases, including 1,185,721 deaths worldwide, as of October 31, 2020.^1^ COVID‐19 is caused by infection from the newly discovered, highly contagious virus, severe acute respiratory syndrome coronavirus 2 (SARS‐CoV‐2).^2^ Severe cases can rapidly progress to a series of syndromes, such as acute respiratory distress syndrome, septic shock, multiple organ dysfunction syndrome, and even death.^3^

While COVID‐19 mainly affects the lungs, cardiac injury is frequently observed by monitoring the levels of high‐sensitivity cardiac troponin I (hs‐TnI) and is reportedly associated with worsened mortality.^4^, ^5^ Cardiovascular complications, such as malignant arrhythmia (atrial fibrillation, ventricular tachycardia, and ventricular fibrillation), myocarditis, and heart failure, all of which can be life‐threatening, are common as well.^6^, ^7^ Systemic inflammation, including sepsis, can reportedly lead to an increased risk of cardiac injury.^8^ On the other hand, angiotensin‐converting enzyme 2 (ACE2), the essential receptor for SARS‐CoV‐2 invasion, is expressed in the cardiovascular system and may lead to direct cardiomyocyte infection.^9^ Thus far, inflammation, hypoxia, and direct virus infection have become the major hypotheses for cardiac involvement in the general population with COVID‐19.^10^ However, the definite mechanism of cardiac injury during COVID‐19 remains unclear. Age has been widely established as a key risk factor for infection and aggravation of COVID‐19. It has been observed that geriatric patients are at a higher risk of poor prognosis after SARS‐CoV‐2 infection; thus, how the virus affects the heart in these patients and how to predict cardiac injury are crucial.^11^ However, studies focusing on cardiac injury in geriatric patients are limited. Hence, we performed a retrograde analysis of cardiac injury in elderly patients to determine the clinical and experimental factors related to such injuries in this population. Our study aimed to reveal the mechanism behind cardiac injury in geriatric patients with COVID‐19 and predict cardiac risk.

## 2 METHODS

### 2.1 Study design and participants

**FIGURE 1:**
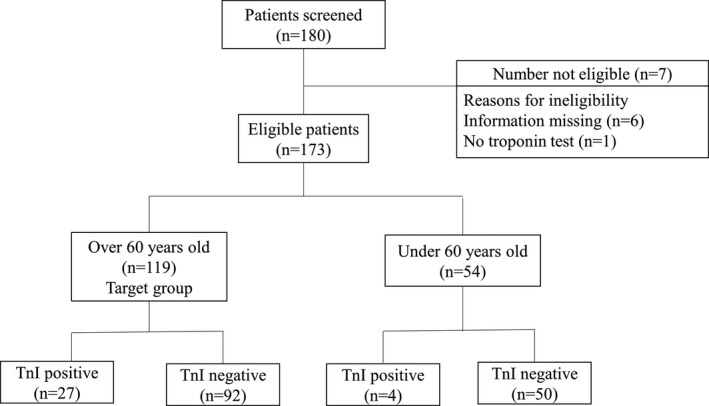
Patient screening and enrollment flow chart

Patients from several treatment centers in Tongji Hospital of Huazhong University of Science and Technology, Wuhan, China were enrolled in this study. All hospitalized patients who were over 60 years of age with confirmed COVID‐19 diagnosis between February 8, 2020 and March 10, 2020 were included. Patients who did not undergo an hs‐TnI test and had incomplete medical records were excluded. Only oral informed consent was obtained, in consideration of an emergency. Of the 180 patients screened, seven did not meet the eligibility criteria (one patient lacked the troponin test and six patients had incomplete information), and 54 patients who were younger than 60 years (youth group) were also excluded (Figure 1). There were 27 patients in the Tnl‐positive group (over the reference interval: men, 34.2 ng/ml; women 15.6 ng/ml) and 92 in the Tnl‐negative group. All diagnoses were confirmed according to the World Health Organization interim guidelines.^12^ All patients had previously undergone a series of tests that included high‐throughput sequencing or real‐time reverse transcriptase polymerase chain reaction (PCR) for nasopharyngeal and anal swabs, computed tomography scanning, and a physical examination.

This study was conducted in accordance with the Declaration of Helsinki. Ethical approval was obtained from the Medical Ethics Committee of the China‐Japan Union Hospital of Jilin University (2020032622) and the Medical Ethics Committee of Tongji Hospital of Huazhong University of Science and Technology (TJ‐IRB20200345).

### 2.2 Data collection

Clinical and laboratory test results were collected from electronic medical records and included symptom presentation (fever, cough, sputum, dyspnea, diarrhea, or chest pain), medical history (coronary heart disease [CHD], hypertension, diabetes, stroke, chronic kidney disease, malignant disease, or chronic obstructive pulmonary disease), cardiac markers (hs‐TnI, creatine kinase‐MB [CK‐MB], myoglobin, and N‐terminal pro‐brain natriuretic peptide [NT‐proBNP]), liver function, serum ions, kidney function, complete blood cell count, arterial blood gas analysis, cytokines, immunity function, lymphocyte subsets, coagulation function, thyroid function, and ferritin levels. Two researchers transferred the data from the patient medical records to Microsoft Excel tables, which were verified by another researcher to ensure their veracity.

### 2.3 Statistical analysis

Statistical analyses were performed using SPSS version 25.0 (IBM Corp., Armonk, NY, USA). Binary variables are described as frequency rates and percentages, and continuous variables are described using median and interquartile range (IQR) values. A portion of the partial continuous data were first converted to binary variables by the defined reference interval because of a difference in the reference interval between sexes. All binary variables were compared using the chi‐square test and continuous variables using Spearman's rank correlation coefficient. The indicators that showed the most significant differences in the single‐factor analysis were assessed by bivariate and multivariate logistic regressions. The odds ratio (OR) with a 95% confidence interval (CI) was also computed and adjusted for age and sex. For all statistical analyses, a *p* value < 0.05 was considered significant.

## 3 RESULTS

### 3.1 Patient characteristics

The study population included 119 hospitalized patients over 60 years of age with a confirmed diagnosis of COVID‐19. The median age was 69 years (IQR: 66–76 years; range: 60–88 years), and 53 patients (44.5%) were men. Patients in the TnI‐positive group were significantly older than those in the TnI‐negative group (median age, 76 years [IQR, 69–82] vs. 69 years [IQR, 65–73.75]; *p* < 0.001). No significant difference was observed in the risk of cardiac injury between men and women (10/53, 18.9% men vs. 17/66, 25.8% women; *p* = 0.372). Fever (98, 82.4%) and cough (81, 68.1%) were the most common symptoms. There were 82 (68.9%) patients with one or more coexisting diseases, with hypertension (60, 50.4%) and diabetes (26, 21.8%) being the most common. However, no statistical significance was observed in symptom presentation and medical history between the TnI‐positive and TnI‐negative groups (Table 1).

**Table 1:** Characteristics of patients with COVID‐19

	No. (%)			*p* value
	Total (*n* = 119)	TnI‐positive (*n* = 27)	TnI‐negative (*n* = 92)	
Age, median (IQR), years	69 (66–76)	76 (69–82)	69 (65–73.75)	<0.001
Sex				
Male	53 (44.5)	10 (37.0)	43 (46.7)	0.372
Female	66 (55.5)	17 (63.0)	49 (53.3)	
Presenting symptom				
Fever	98 (82.4)	22 (81.5)	76 (82.6)	0.893
Cough	81 (68.1)	17 (63.0)	64 (69.6)	0.518
Sputum	40 (33.6)	11 (40.7)	29 (31.5)	0.373
Dyspnea	48 (40.3)	12 (44.4)	36 (39.1)	0.621
Diarrhea	37 (31.1)	7 (26.0)	30 (32.6)	0.509
Chest pain	9 (7.6)	2 (7.4)	7 (7.6)	0.972
Medical history				
CHD	19 (16.0)	3 (11.1)	16 (17.4)	0.418
Hypertension	60 (50.4)	15 (55.6)	45 (48.9)	0.544
Diabetes	26 (21.8)	6 (22.2)	20 (21.7)	0.957
Stroke	5 (4.2)	2 (7.4)	3 (3.3)	0.318
CKD	4 (3.4)	1 (3.7)	3 (3.3)	1
Malignant disease	7 (5.9)	1 (3.7)	6 (6.5)	0.565
COPD	2 (1.7)	1 (3.7)	1 (1.1)	0.404

Abbreviations: CHD, coronary heart disease; CKD, chronic kidney disease; COPD, chronic obstructive pulmonary disease.

* *p* values indicate differences between TnI‐positive and TnI‐negative groups; *p* < 0.05 was considered statistically significant.

**FIGURE 2:**
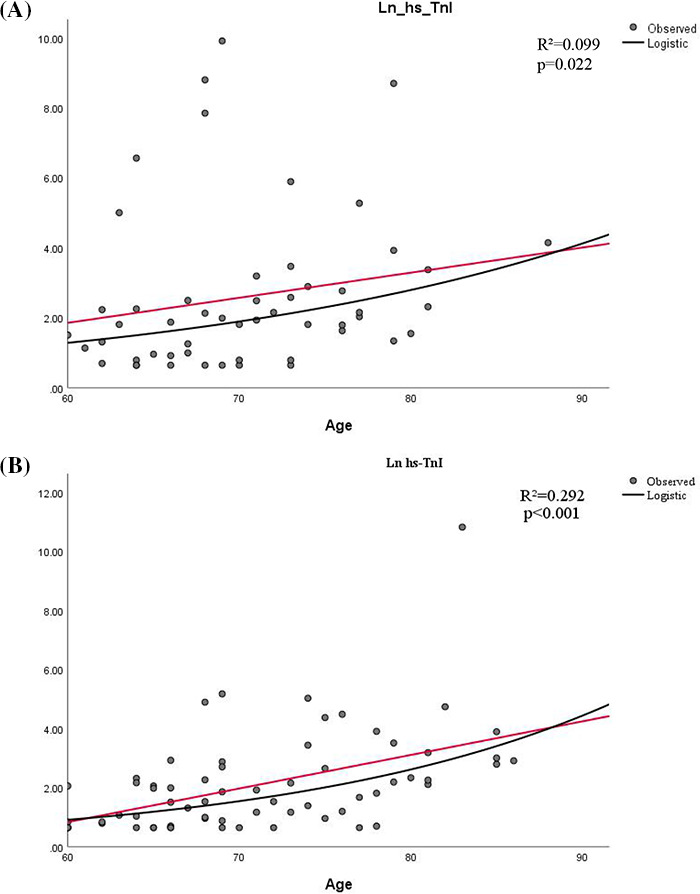
Correlation between age and Ln hs‐TnI. A, Correlation between age and Ln hs‐TnI in male patients. B, Correlation between age and Ln hs‐TnI in female patients. Ln hs‐TnI, natural logarithm of high‐sensitivity cardiac troponin. Black line: age; red line: fitted line

Curve estimation analysis using hs‐TnI as a continuous variable demonstrated that age correlated positively with the natural logarithm of hs‐TnI levels (men, *R* ^2^ = 0.099, *p* = 0.022; women, R^2^ = 0.292, *p* < 0.001). The curves for male and female patients were plotted separately (Figure 2).

### 3.2 Laboratory findings

The analysis of all laboratory tests and all reference intervals are shown in Table 2. Of 119 patients, hs‐TnI levels were elevated in 27 (22.7%) patients and were over four times elevated in 15 (12.6%) patients during hospitalization, with a significantly higher rate than in the youth group (4 in 54, 7.4%). Compared to the corresponding levels in the TnI‐negative group, myoglobin (*p* < 0.001), CK‐MB (*p* = 0.001), NT‐proBNP (*p* < 0.001), creatine kinase (CK) (*p* = 0.007), D‐dimer (*p* < 0.001), and lactate dehydrogenase (*p* < 0.001) levels were significantly higher in the positive group.

**Table 2:** Laboratory findings of patients with COVID‐19

	Reference interval	Median (IQR)			*p* value
		Total	TnI‐positive	TnI‐negative	
		(*n* = 119)	(*n* = 27)	(*n* = 92)	
hs‐TnI, pg/ml	Men, ≤34.2	6.9 (2.65–21.15)	533.75 (127.125–6134.825)	6 (2.2–9.3)	N/A
	Women, ≤15.6	6.25 (2.3–16.65)	48.9 (19.35–123.4)	3.2 (1.95–7.55)	
Myoglobin, ng/ml	Men, ≤154.9	87.3 (44–134.6)	192.4 (122.725–248.225)	64.3 (40.9–119.8)	<0.001*
	Women, ≤106	38.2 (27.7–63.775)	120.9 (65.5–265.325)	34.1 (26.825–54.125)	
CK‐MB, ng/ml	Men, ≤7.2	1.2 (0.7–1.9)	4.05 (1.625–11.85)	1 (0.6–1.5)	0.001*
	Women, ≤3.4	0.7 (0.35–1.2)	1.95 (0.9–9.425)	0.6 (0.3–0.8)	
NT‐proBNP, pg/ml	<241	221 (113–707)	1371 (543–3304)	176 (96.25–344.5)	<0.001*
WBC count, ×10^9^/L	3.5–9.5	5.85 (4.49–7.27)	7.76 (6.19–10.19)	5.505 (4.3825–6.84)	<0.001*
Neutrophil, %	40–75	71 (60.8–81.5)	83.4 (75.1–87.1)	67.45 (58.825–76.5)	<0.001*
Neutrophil count, ×10^9^/L	1.8–6.3	4.02 (2.81–5.63)	7.23 (4.46–9.01)	3.835 (2.5525–4.7725)	<0.001*
Lymphocyte, %	20–50	17.5 (10.9–28.2)	8.7 (5.4–15.9)	19.95 (13.925–29.5)	<0.001*
Lymphocyte count, ×10^9^/L	1.1–3.2	1.02 (0.69–1.41)	0.81 (0.59–1.1)	1.1 (0.735–1.515)	<0.001*
Monocyte, %	3.0–10.0	8.7 (6.5–10.1)	6.2 (4.5–8.7)	9.1 (7.5–10.275)	<0.001*
Monocyte count, ×10^9^/L	0.1–0.6	0.54 (0.39–0.66)	0.5 (0.34–0.78)	0.54 (0.39–0.64)	0.714
Eosinophil, %	0.4–8	0.6 (0.2–1.7)	0.3 (0–1.1)	0.75 (0.2–1.8)	0.050
Eosinophil count, ×10^9^/L	0.02–0.52	0.04 (0.01–0.11)	0.03 (0–0.09)	0.05 (0.01–0.11)	0.386
Basophil, %	0–1	0.2 (0.1–0.3)	0.2 (0.1–0.3)	0.2 (0.1–0.375)	0.217
Basophil count, ×10^9^/L	0–0.1	0.01 (0.01–0.02)	0.01 (0.01–0.03)	0.01 (0.01–0.02)	0.139
Erythrocyte count, ×10^12^/L	4.3–5.8	3.96 (3.61–4.41)	4.08 (3.61–4.67)	3.955 (3.595–4.38)	0.299
Hemoglobin, g/L	130–175	123 (110–134)	126 (102–136)	121 (111–133)	0.686
Platelet count, ×10^9^/L	125–350	240 (168–292)	188 (105–253)	241.5 (184.5–296.75)	0.012*
ESR, mm/H	0–15	41 (22–68.5)	35 (16–56)	48 (25–70.5)	0.084
PT, s	11.5–14.5	14.1 (13.5–14.6)	14.6 (13.8–15.6)	14 (13.475–14.4)	0.003*
PTA, %	75–125	89 (83–97.5)	81 (73–91)	90 (85–99)	0.001*
INR, µmol/L	0.8–1.2	1.07 (1.015–1.125)	1.14 (1.06–1.22)	1.065 (1.01–1.11)	0.002*
Fibrinogen, g/L	2.0–4.0	5.1 (3.955–6.175)	5.3 (3.92–5.99)	5.08 (3.9625–6.185)	0.857
APTT, s	29–42	39.6 (36.4–42.95)	39.6 (36.4–43.5)	39.85 (36.25–42.475)	0.852
TT, s	14–19	16.8 (15.8–17.75)	16.9 (15.5–17.8)	16.75 (15.875–17.65)	0.824
D‐dimer, μg/ml FEU	<0.5	1.43 (0.6–2.745)	3.71 (1.19–21)	1.185 (0.545–2.205)	<0.001*
FDPs, μg/ml	<5	5 (4–14.1)	15.9 (4.25–63.65)	4.4 (4–7.8)	0.004*
Antithrombin, %	80–120	91 (83–105.75)	91 (79–106)	91 (84–105)	0.718
ALT, U/L	Men, ≤41	26 (18–41)	31 (18.25–43)	24 (17–40)	0.648
	Women, ≤33	17 (12–29.25)	16 (12–33)	17 (12–28)	
AST, U/L	Men, ≤40	27 (20–37)	31 (24.5–49.5)	25 (19–35)	0.648
	Women, ≤32	21.5 (16.75–32.25)	25 (19–36.5)	21 (16–32.5)	
Total protein, g/L	64–83	68.3 (64.4–72.4)	68.2 (64.4–70.8)	68.35 (64.275–72.55)	0.638
Albumin, g/L	35–52	33.4 (30.9–37.2)	32.3 (30.2–35.3)	34.4 (30.9–37.7)	0.161
Globulin, g/L	20–35	34.2 (30.4–37)	36.3 (31.8–37.6)	33.25 (30.4–36.775)	0.143
Prealbumin, mg/L	200–400	202 (116–243)	216.5 (115.25–280)	201 (115.5–242)	0.489
TBil, µmol/L	Men, ≤26	10.9 (8.7–14.8)	11.85 (8.875–24.45)	10.6 (8.7–14.6)	1.000
	Women, ≤21	10.25 (7.45–14.025)	11 (8.95–16.2)	10 (7.15–12.6)	
DBil, µmol/L	≤8	4.6 (3.2–6.3)	6.2 (3.4–8.9)	4.3 (3.125–5.775)	0.026*
IBil, µmol/L	Men, ≤16.8	5.9 (4.55–8.4)	7.1 (4.225–10.425)	5.8 (4.5–8.2)	0.541
	Women, ≤12.9	5.6 (4.275–8)	5.5 (3.85–8.4)	6 (4.35–7.8)	
ALP, U/L	Men, 40–130	69 (58.5–83.5)	80.5 (68.5–102.75)	65 (54–80)	0.646
	Women, 35–105	65 (54.75–87)	67 (50.5–79.5)	63 (55.5–88)	
γ‐glutamyl transpeptidase, U/L	Men, 10–71	28 (20.5–58)	40.5 (20.5–87.5)	27 (20–47)	0.431
	Women, 6–42	19.5 (15–46.75)	20 (17–40.5)	19 (14.5–48.5)	
Total cholesterol, mmol/L	<5.18	3.9 (3.15–4.44)	4.02 (3.07–4.6)	3.895 (3.175–4.4075)	0.990
Triglyceride, mmol/L	<1.7	1.18 (0.93–1.69)	1.335 (1.07–1.7825)	1.15 (0.92–1.69)	0.125
HDL, mmol/L	1.04–1.55	0.94 (0.77–1.07)	0.94 (0.8075–1.165)	0.94 (0.765–1.06)	0.666
LDL, mmol/L	<3.37	2.37 (1.87–2.93)	2.25 (1.5525–2.6075)	2.37 (1.87–2.995)	0.130
CK, U/L	Men, ≤190	80 (55.25–119.75)	205.5 (76.75–271.75)	70.5 (55.25–96.75)	0.007*
	Women, ≤170	49 (36–84.5)	37 (27.5–137)	51 (37.5–78.5)	
LDH, U/L	135–225	269 (214–364)	384 (245–646)	259 (206.75–311)	<0.001*
K, mmol/L	3.5–5.1	4.33 (3.93–4.75)	4.27 (3.68–5.02)	4.34 (3.985–4.7375)	0.835
Na, mmol/L	136–145	140.1 (138–142.4)	140.2 (137.8–142.2)	140 (138.025–142.55)	0.980
Cl, mmol/L	99–110	101.2 (98.3–103.6)	99.7 (97.4–104.4)	101.3 (98.375–103.375)	0.796
Ca, mmol/L	2.2–2.55	2.15 (2.07–2.25)	2.15 (2.06–2.23)	2.155 (2.07–2.25)	0.686
P, mmol/L	0.81–1.45	1.12 (0.86–1.25)	1.26 (0.935–1.68)	1.09 (0.86–1.23)	0.087
Mg, mmol/L	0.66–0.99	0.85 (0.79–0.91)	0.815 (0.7725–1.025)	0.87 (0.81–0.91)	0.867
Urea, mmol/L	Men, 3.6–9.5	5.6 (4.1–8.5)	11.4 (9.5–15.3)	4.9 (3.7–6.5)	<0.001*
	Women, 3.1–8.8	4.15 (3.1–5.55)	7.4 (5.35–15.05)	3.4 (3–4.6)	
Creatinine, µmol/L	Men, 59–104	82 (69.5–93)	93.5 (83.75–165)	75 (66–91)	<0.001*
	Women, 45–84	64 (56.75–75.25)	80 (64–99)	60 (55.5–69)	
Trioxypurine, µmol/L	Men, 202.3–416.5	264 (207.5–303)	313.5 (257.75–403.5)	255 (183–295)	0.003*
	Women, 142.8–339.2	261.5 (172.75–297)	323 (227.5–461)	255 (169.5–280.5)	
HCO^−^, mmol/L	22–29	24.8 (23.1–27)	23.8 (21.5–25.4)	25.15 (23.35–27.1)	0.022*
Total bile acid, µmol/L	≤10	4.6 (2.9–6.95)	5.1 (3.15–6.4)	4.5 (2.9–7.05)	0.987
a‐L‐fucosidase, IU/L	5–40	22 (18–27)	22 (16.75–28.5)	22 (18–27)	0.974
Cholinesterase, U/L	5320–12920	6448 (4752.25–7499.5)	4188.5 (3345.25–8062.75)	6628.5 (5188–7484.25)	0.245
Cystatin C, mg/L	0.6–1.55	1.03 (0.92–1.405)	2.645 (1.1–5.0225)	1 (0.905–1.21)	0.004*
Total amylase, U/L	28–100	63 (48–75.75)	71 (60.5–106.5)	62 (45.75–75.25)	0.134
eGFR, ml/min/1.73 m^2^	>90	85.2 (69.3–92.8)	67.1 (48.2–80.7)	89.4 (74.925–94.3)	<0.001*
Procalcitonin, ng/ml	<0.05	0.05 (0.03–0.1225)	0.205 (0.095–0.3375)	0.03 (0.0225–0.06)	<0.001*
IL‐1β, pg/ml	<5	5 (5–5)	5 (5–5)	5 (5–5)	0.218
IL‐2R, U/ml	223–710	602.5 (373–1012.25)	1062 (593–1646.5)	541 (353.5–869.5)	<0.001*
IL‐6, pg/ml	<7	7.25 (1.93–28.8)	24.38 (6.145–44.575)	4.875 (1.57–17.13)	0.004*
IL‐8, pg/ml	<62	9.45 (5–19.525)	10.7 (6.1–28.35)	9 (5–17.9)	0.129
IL‐10, pg/ml	<9.1	5 (5–5)	5 (5–6.2)	5 (5–5)	0.047*
TNF‐α, pg/ml	<8.1	6.9 (4.775–10.625)	10.3 (5.4–14.25)	6.6 (4.4–9.3)	0.018*
hs‐CRP, mg/ml	<1	20 (3.5–75.1)	65.8 (10.3–131.1)	15.85 (2.975–56.225)	0.005*
Total T (CD3+ CD19−), %	50–84	73.985 (63.13–78.9525)	74.3 (63.13–80.245)	73.85 (62.3–78.72)	0.660
Total T (CD3+ CD19−) count,/μl	955–2860	973.5 (784.5–1166)	873 (542.5–1139.5)	974 (786–1185)	0.391
Total B (CD3− CD19+), %	5–18	12.17 (8.955–16.685)	16.74 (9.545–25.045)	12.04 (8.04–16.05)	0.146
Total B (CD3− CD19+) count,/μl	90–560	169.5 (112.5–255.5)	198 (121–245.5)	167 (102–271)	0.700
Helper T (CD3+ CD4+), %	27–51	45.91 (37.8225–49.7825)	48.94 (44.22–52.675)	44.81 (37.54–49.4)	0.114
Helper T (CD3+ CD4+) count,/μl	550–1440	600 (478–762.75)	554 (351.5–708)	616 (490–802)	0.507
Suppressor T (CD3+ CD8+), %	15–44	22.23 (17.215–28.31)	22.89 (16.385–25.725)	22.17 (17.23–29.99)	0.487
Suppressor T (CD3+ CD8+) count,/μl	320–1250	282 (243.5–385.5)	268 (180.5–348)	300 (243–388)	0.299
NK cell (CD3−/CD16+ CD56+), %	7–40	13.015 (9.385–18.6075)	9.43 (7.77–12.07)	13.74 (9.61–19.89)	0.004*
NK cell (CD3−/CD16+ CD56+) count,/μl	150–1100	176 (117.5–273.25)	108 (54–181)	197 (135–313)	0.001*
Th/Ts	0.71–2.78	2.025 (1.405–2.6075)	2.14 (1.855–2.865)	1.94 (1.36–2.6)	0.179
Ig A, g/L	0.82–4.53	2.115 (1.6475–3.1675)	2.725 (2.1325–3.515)	1.98 (1.5625–2.6325)	0.081
Ig G, g/L	7.51–15.6	11.2 (9.325–13.375)	12.35 (9.5–14.55)	10.95 (9.275–13.15)	0.366
Ig M, g/L	0.46–3.04	0.87 (0.62–1.09)	0.925 (0.585–1.1225)	0.86 (0.6125–1.1)	0.883
C3, g/L	0.65–1.39	0.87 (0.73–0.95)	0.915 (0.745–0.9675)	0.87 (0.7275–0.9525)	0.776
C4, g/L	0.16–0.38	0.24 (0.1825–0.29)	0.255 (0.1925–0.32)	0.24 (0.18–0.29)	0.371
PH	7.35–7.45	7.422 (7.391–7.4545)	7.446 (7.415–7.476)	7.411 (7.38525–7.44575)	0.062
paCO_2_, mmHg	35–45	39.8 (35.75–43.4)	38.3 (30.3–42.8)	40.5 (37.075–43.65)	0.368
paO_2_, mmHg	80–100	136 (89.55–193)	105 (85.9–176)	150 (96.35–205.5)	0.094
AB, mmol/L	21–28	24.7 (23.45–26.55)	24.7 (21.5–27.5)	24.85 (23.5–26.4)	0.896
SB, mmol/L	21–25	25.3 (23.925–27.075)	25.3 (23.8–28)	25.3 (23.95–26.6)	0.840
BEb, mmol/L	−3–+3	0.9 (−0.6–3)	1 (−0.6–4)	0.9 (−0.65–2.55)	0.749
BE‐ECF, mmol/L	−3–+3	0.8 (−0.55–3.05)	0.8 (−1.5–3.9)	0.75 (−0.525–2.525)	0.961
TCO, mmol/L	24–32	22.75 (20.9–24.3)	22.9 (19.5–24.8)	22.7 (21.1–24.25)	0.906
spO_2_, %	91.9–99	99.3 (97.2–99.65)	98.5 (96.8–99.5)	99.35 (97.7–99.725)	0.187
Glucose, mmol/L	4.11–6.05	5.98 (5.16–7.26)	6.1 (5.29–8.07)	5.875 (5.16–7.1475)	0.514
Ferritin, μg/L	Men, 30–400	666.4 (420.6–1263.3)	975.7 (633.45–1736.825)	653.8 (362.1–1212.7)	0.352
	Women, 15–150	436.45 (264.9–731.825)	748.5 (246.5–1461.2)	428.1 (300.7–654.95)	

Note

All data with sex differences were converted to binary variables before analysis.

Abbreviations: AB, actual bicarbonate; ALP, alkaline phosphatase; ALT, alanine aminotransferase; APTT, activated partial thromboplastin time; AST, aspartate aminotransferase; BEb, base excess blood; BE‐ECF, base excess extracellular fluid; CK, creatine kinase; CK‐MB, creatine kinase isoenzyme MB; DBil, direct bilirubin; eGFR, estimated glomerular filtration rate; ESR, erythrocyte sedimentation rate; FDPs, fibrin degradation products; HDL, high‐density lipoprotein; hs‐CRP, high‐sensitive C‐reaction protein; hs‐CRP, high‐sensitivity cardiac troponin I; IBil, indirect bilirubin; IL, interleukin; INR, international normalized ratio; LDH, lactic dehydrogenase; LDL, low‐density lipoprotein; NT‐proBNP, N‐terminal pro‐brain natriuretic peptide; PT, prothrombin time; PTA, prothrombin activity; SB, standard bicarbonate; TBil, total bilirubin; TCO, total CO_2_; TNF‐α, tumor necrosis factor; TT, thrombin time; WBC, white blood cell.

* *p* values indicate differences between TnI‐positive and TnI‐negative groups; *p* < .05 was considered statistically significant.

In addition to cardiovascular markers, a wealth of data showed significant differences between the two groups. For routine hematological indices, white blood cells (WBCs) (*p* < 0.001) and neutrophils (*p* < 0.001) were higher in the positive group and lymphocytes (*p* < 0.001) showed a marked decline, which was attributed solely to a decline in the proportion of monocytes (*p* < 0.001). It is worth noting that significant differences were observed in several inflammatory markers (high‐sensitivity C‐reactive protein [hs‐CRP] [*p* = 0.005], procalcitonin [PCT] [*p* < 0.001], interleukin‐2 receptor [IL‐2R] [*p* < 0.001], interleukin 6 [IL‐6] [*p* = 0.004], interleukin 10 [IL‐10] [*p* = 0.047], tumor necrosis factor α [TNF‐α] [*p* = 0.018]), and immunological markers (natural killer cells [NK cells] [CD3‐/CD16+, CD56+] [*p* = 0.004]).

Meanwhile, we found that creatinine (*p* < 0.001), trioxypurine (*p* = 0.003), cystatin C (*p* = 0.004), and estimated glomerular filtration rate (eGFR) (*p* < 0.001) values in the positive group significantly differed compared to those in the negative group, and this suggests that renal injury may be related to cardiac injury in patients with COVID‐19.

### 3.3 Factors associated with cardiac injury

**FIGURE 3:**
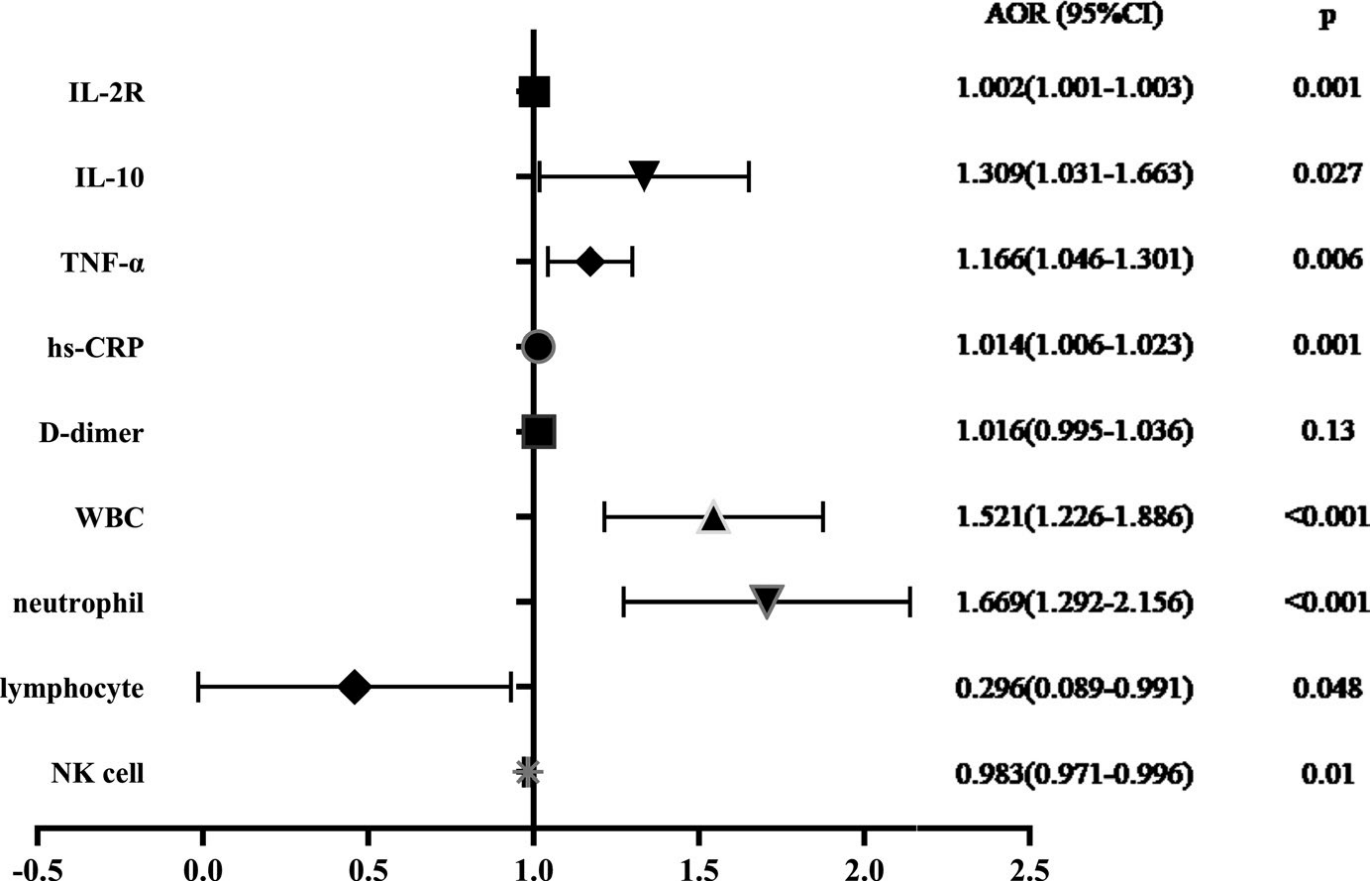
Forest map of the risk factors associated with cardiac injury. AOR, adjusted odds ratio; CI, confidence interval; IL‐2, interleukin‐2; IL‐10, interleukin 10; TNF‐α, tumor necrosis factor α; hs‐CRP, high‐sensitivity C‐reactive protein; WBC, white blood cell; NK cell, natural killer cell

We used logistic regression to examine the factors relevant to cardiac injury. Variables that were considered to be potential risk factors and showed statistical significance in the single‐factor analysis were subjected to bivariate logistic regression and then adjusted for age and sex (Table 3; Figure 3).

**Table 3:** Logistic regression analysis of factors associated with cardiac injury

					AGE	
	Crude OR (95% CI)	*p* value	Adjusted OR (95% CI)	*p* value	Adjusted OR (95% CI)	*p* value
PCT	7.474 (2.384–23.436)	0.001	8.65 (2.433–30.752)	0.001*	1.146 (1.056–1.243)	0.001
IL‐2R	1.001 (1–1.002)	0.002	1.002 (1.001–1.003)	0.001*	1.178 (1.08–1.286)	<0.001
IL‐6	3.214 (1.222–8.454)	0.018	3.724 (1.186–11.689)	0.024*	1.144 (1.059–1.235)	0.001
IL‐10	1.226 (0.993–1.514)	0.058	1.309 (1.031–1.663)	0.027*	1.162 (1.075–1.257)	<0.001
TNF‐α	1.152 (1.048–1.265)	0.003	1.166 (1.046–1.301)	0.006*	1.144 (1.058–1.238)	0.001
hs‐CRP	1.01 (1.003–1.017)	0.003	1.014 (1.006–1.023)	0.001*	1.175 (1.083–1.275)	0.001
D‐dimer	1.007 (0.988–1.027)	0.462	1.016 (0.995–1.036)	0.13	1.171 (1.084–1.266)	<0.001
WBC	1.413 (1.191–1.677)	<0.001	1.521 (1.226–1.886)	<0.001*	1.173 (1.075–1.28)	<0.001
Neutrophil	1.499 (1.24–1.813)	<0.001	1.669 (1.292–2.156)	<0.001*	1.173 (1.071–1.285)	0.001
Lymphocyte	0.309 (0.114–0.836)	0.021	0.296 (0.089–0.991)	0.048*	1.147 (1.063–1.239)	<0.001
NK cell	0.987 (0.977–0.996)	0.008	0.983 (0.971–0.996)	0.01*	1.213 (1.06–1.389)	0.005

Note

PCT and IL‐6 were first converted to binary variables due to improper data distribution.

Age was analyzed as an adjustment factor, and the *p* value of age represented the statistical significance of age in the logistic regression model.

* *p* values indicate differences between TnI‐positive and TnI‐negative groups; *p* < .05 was considered statistically significant.

Inflammatory mediators yielded significant results in the analysis. Patients with a positive PCT level (≥0.05 ng/ml) were nearly eight times more likely to develop cardiac injury than patients with a negative PCT level (adjusted OR [AOR]: 8.65; 95% CI: 2.433–30.752; *p* = 0.001). Patients with positive IL‐6 (≥7 pg/ml) showed an almost four‐fold greater risk than those with negative IL‐6 (AOR: 3.724; 95% CI: 1.186–11.689; *p* = 0.024); a 1 pg/ml increase in TNF‐α resulted in a 16.6% increased risk (AOR, 1.166; 95% CI, –1.046 to 1.301; *p* = 0.006), and a 1 pg/ml increase in hs‐CRP resulted in a 1.4% increased risk (AOR: 1.014; 95% CI: 1.006–1.023; *p* = 0.001) of cardiac injury.

WBC (AOR: 1.521; 95% CI: 1.226–1.886; *p* < 0.001) and neutrophil levels (AOR: 1.669; 95% CI: 1.292–2.156; *p* < 0.001) were closely related to cardiac injury. Furthermore, the analysis revealed descending predicted probabilities as lymphocytes (AOR: 0.296; 95% CI: 0.089–0.991; *p* = 0.048) and NK cells (AOR, 0.983; 95% CI, –0.971 to 0.996; *p* = 0.01) increased in elderly patients with COVID‐19. For each unit (count: 10^9^/L) increase in lymphocyte count, the odds of cardiac injury decreased from 1 to 0.296.

However, D‐dimer (AOR: 1.016; 95% CI: 0.995–1.036; *p* = 0.13) resulted in no statistical significance in the logistic regression. This may be caused by individual outliers, as the D‐dimer test results changed after eliminating a single case (OR, 1.175; 95% CI, –1.083 to 1.275; *p* < 0.001).

## 4 DISCUSSION

Despite previous studies on cardiac injury in patients with COVID‐19, few analyses have investigated cardiac injury specifically in the high‐risk elderly population. Our study analyzed specific serological information from the viewpoint of assessing the role of serological markers in predicting cardiac injury in elderly patients with COVID‐19. The majority of the included patients had different coexisting diseases, but no statistically significant difference was observed between the TnI‐positive and TnI‐negative groups. We identified a range of indicators that showed significant differences as hs‐TnI values increased, including coagulation indicators, peripheral blood cells, and inflammatory cytokines. For the 27 positive cases, the most significant indicators of abnormality were deviations in the D‐dimer (27 in 27, 100%), hs‐CRP (26 in 27, 96.3%), PCT (24 in 27, 88.9%), lymphocytes (24 in 27, 88.9%), neutrophils (21 in 27, 77.8%), IL‐6 (18 in 25, 72%), IL‐2R (16 in 25, 64%), NK cells (8 in 13, 61.5%), TNF‐α (15 in 25, 60%), and WBC (12 in 27, 44.4%) values. Consistently, most prior reports have indicated that a considerable proportion of patients had varying degrees of cardiac injury, especially those with more severe COVID‐19. A rising TnI level is a predictive factor for poor clinical outcomes. Ni et al indicated that acute cardiac injury was observed in 41% of non‐survivors with COVID‐19 at initial hospitalization.^13^ It is extensively recognized that cardiac injury plays an important role in the outcome of COVID‐19. However, owing to the limitations of research on SARS‐CoV‐2 with respect to animal experimentation, the mechanisms of cardiac injury remain unclear.

With progress in the research on COVID‐19, age has been widely accepted as a significant risk factor for infection and disease aggravation. Sun et al reported that patients with COVID‐19 were significantly older than individuals with negative SARS‐CoV‐2 PCR results.^14^ Several studies have demonstrated that elderly patients with COVID‐19 have a much higher probability of worsening conditions.^3^, ^11^ In our study, 22.7% (27/119) of patients in the elderly group were observed to have cardiac injury, an incidence rate higher than that seen in the youth group (7.4%, 4/54), and this indicates that age plays an important role in cardiac injury in patients with COVID‐19. On the other hand, elderly patients have a higher risk of experiencing other diseases that may lead to chronic inflammation and elevated inflammatory cytokine levels. Our analysis revealed that coexisting diseases, including hypertension and CHD, were not statistically correlated with cardiac injury, although this may be due to the small sample size of our study. In the logistic regression model, age was shown to have statistical significance (Table 3); every 1‐year increase in age was associated with at least a 14.4% rise in cardiac injury risk.

Severe acute pneumonia, such as in COVID‐19, can be roughly divided into three periods: virus amplification, excessive immune response, and recovery, exacerbation, or even death.^15^ In the hs‐TnI‐positive patients in our study, WBC and neutrophil counts significantly increased, whereas lymphocyte, NK cell, and monocyte counts decreased. Inflammatory mediators, including PCT, IL‐2R, IL‐6, IL‐10, TNF‐α, and hs‐CRP, also showed marked abnormalities. This noteworthy inflammation in patients with COVID‐19 and significant changes in the numbers of inflammatory cells and mediators were also observed in other studies, especially severe changes known as a cytokine storm,^16^, ^17^ which Li et al referred to as “viral sepsis.”^18^ Investigations of bronchoalveolar lavage fluid by Zhou et al demonstrated that hypercytokinemia and pro‐inflammatory pathways were mediated by interleukins and TNF‐α in patients with COVID‐19.^19^ Several autopsy reports of patients with COVID‐19 have also revealed neutrophil and monocyte infiltration in heart tissue.^20^, ^21^ Furthermore, several pathways associated with both aging and inflammation have been identified in patients with COVID‐19, such as age‐related redox imbalance, autophagy slowing, and senescent cells.^22^ These pathways trigger the inflammasome and lead to an inflammatory cascade.

The logistic regression analysis results revealed that inflammatory markers had a strong predictive ability for cardiac injury in patients with COVID‐19. The possible mechanisms include cytokine storm, fulminant myocarditis, direct damage by SARS‐CoV‐2, type 2 myocardial infarction caused by dyspnea, and microcirculation disturbance caused by inflammation. The strong relationship between systemic inflammation and cardiac injury has been proven previously; mediators such as TNF, toll‐like receptor 4, neutrophil‐to‐lymphocyte ratio, and neutrophil extracellular traps are considerably important in cardiac injury.^23^, ^24^, ^25^ A clinical study by Elissa et al reported that 7% of COVID‐19 deaths were caused by myocarditis.^26^ However, this was only estimated by experience and not substantiated by a confirmed diagnosis. Recent research has demonstrated that the use of angiotensin receptor blockers and ACE inhibitors did not increase the risk of COVID‐19 infection or aggravation. More convincingly, in patients with diabetes, a decreased risk of COVID‐19 requiring hospitalization was observed in users of renin‐angiotensin‐aldosterone system inhibitors.^27^ Therefore, we speculated that systemic inflammation and the subsequent cytokine storm are the major risk factors for cardiac injury in patients with COVID‐19.

For the excessive inflammatory response observed during COVID‐19 infection, proper pharmaco‐immunomodulating strategies may help improve patient condition. Several cytokine antagonists have been proven to be potential therapeutics, including IL‐1 receptor antagonists, IL‐6 receptor antagonists, and anti‐TNF‐α.^28^ A clinical study by Fernández‐Ruiz et al also indicated that tocilizumab, an anti‐IL‐6 receptor monoclonal antibody, was useful for resolving inflammation and improving patients' clinical condition.^29^ Interfering with inflammatory processes should be as important as blocking virus amplification and may potentially enable cardiac protection.

The elderly patient population is greatly affected by COVID‐19, and cardiac injury is common in patients with COVID‐19 and is closely related to a worse prognosis, which warrants more attention to identify the related factors to continuously monitor the status of elderly patients and guide treatment. Our study suggests a potential relationship between cardiac injury and inflammation in elderly patients with COVID‐19. However, the currently available evidence is inconclusive, and extensive studies on the detailed mechanism of COVID‐19 and cardiac injury are needed to identify their relationship. Some limitations are inevitable at this stage of the COVID‐19 outbreak. First, the sample size was not large enough; thus, we could only provide implied conclusions and contribute to future meta‐analyses and systemic reviews. Second, a lack of temporal monitoring of the inflammatory factors, owing to the retrograde study design, indicates that further research is needed in the future to determine the dynamic changes between inflammatory factors and cardiac injury.

## 5 CONCLUSIONS

Our results suggest that age and inflammatory factors influence cardiac injury in elderly patients. Interfering with inflammation in this patient population may potentially confer cardiac protection.

## CONFLICT OF INTEREST

The authors declare that they have no conflict of interest.

## ETHICAL APPROVAL

This study was conducted in accordance with the Declaration of Helsinki. Ethical approval was obtained from the Medical Ethics Committee of the China‐Japan Union Hospital of Jilin University (2020032622) and the Medical Ethics Committee of Tongji Hospital of Huazhong University of Science and Technology (TJ‐IRB20200345).

## CONSENT TO PARTICIPATE

Only oral informed consent was obtained on consideration of emergency.

## Data Availability

The data that support the findings of this study are available from the corresponding authors upon reasonable request.

## References

[1] World Health Organization . Coronavirus Disease (COVID‐19) Dashboard. https://covid19.who.int/. Accessed 31 October 2020.

[2] Wu Z , McGoogan JM . Characteristics of and important lessons from the coronavirus disease 2019 (COVID‐19) outbreak in China: Summary of a report of 72 314 cases from the Chinese Center for Disease Control and Prevention. JAMA. 2020;323(13):1239‐1242. 32091533 10.1001/jama.2020.2648

[3] Wang D , Hu B , Hu C , et al. Clinical characteristics of 138 hospitalized patients with 2019 novel coronavirus‐infected pneumonia in Wuhan, China. JAMA. 2020;323(11):1061‐1069. 32031570 10.1001/jama.2020.1585 PMC7042881

[4] Hu L , Chen S , Fu Y , et al. Risk factors associated with clinical outcomes in 323 COVID‐19 hospitalized patients in Wuhan, China. Clin Infect Dis. 2020. 10.1093/cid/ciaa539 PMC7197620 32361738

[5] Bonow RO , Fonarow GC , O'Gara PT , Yancy CW . Association of Coronavirus Disease 2019 (COVID‐19) with myocardial injury and mortality. JAMA Cardiol. 2020;5(7):751‐753. 32219362 10.1001/jamacardio.2020.1105

[6] Guzik TJ , Mohiddin SA , Dimarco A , et al. COVID‐19 and the cardiovascular system: implications for risk assessment, diagnosis, and treatment options. Cardiovasc Res. 2020;116(10):1666‐1687. 32352535 10.1093/cvr/cvaa106 PMC7197627

[7] Babapoor‐Farrokhran S , Rasekhi RT , Gill D , Babapoor S , Amanullah A . Arrhythmia in COVID‐19. SN Compr Clin Med. 2020;2(9):1430‐1435. 32838188 10.1007/s42399-020-00454-2 PMC7426193

[8] Gualandro DM , Puelacher C , Mueller C . High‐sensitivity cardiac troponin in acute conditions. Curr Opin Crit Care. 2014;20(5):472‐477. 25159476 10.1097/MCC.0000000000000132

[9] Hoffmann M , Kleine‐Weber H , Schroeder S , et al. SARS‐CoV‐2 cell entry depends on ACE2 and TMPRSS2 and is blocked by a clinically proven protease inhibitor. Cell. 2020;181(2):271.e278‐280.e278. 32142651 10.1016/j.cell.2020.02.052 PMC7102627

[10] Wei ZY , Geng YJ , Huang J , Qian HY . Pathogenesis and management of myocardial injury in coronavirus disease 2019. Eur J Heart Fail. 2020. 10.1002/ejhf.1967 PMC7405025 32683753

[11] Shi M , Chen L , Yang Y , et al. Analysis of clinical features and outcomes of 161 patients with severe and critical COVID‐19: A multicenter descriptive study. J Clin Lab Anal. 2020;34:e23415. 32488958 10.1002/jcla.23415 PMC7300573

[12] World Health Organization . Clinical management of Severe acute respiratory infection when novel coronavirus (nCoV) infection is suspected: interim guidance. https://apps.who.int/iris/handle/10665/330854. Accessed 25 January 2020.

[13] Ni W , Yang X , Liu J , et al. Acute myocardial injury at hospital admission is associated with all‐cause mortality in COVID‐19. J Am Coll Cardiol. 2020;76:124‐125. 32407771 10.1016/j.jacc.2020.05.007 PMC7213968

[14] Sun Y , Koh V , Marimuthu K , et al. Epidemiological and Clinical Predictors of COVID‐19. Clin Infect Dis. 2020;71:786‐792. 32211755 10.1093/cid/ciaa322 PMC7542554

[15] Wong CK , Lam CW , Wu AK , et al. Plasma inflammatory cytokines and chemokines in severe acute respiratory syndrome. Clin Exp Immunol. 2004;136(1):95‐103. 15030519 10.1111/j.1365-2249.2004.02415.x PMC1808997

[16] Qin C , Zhou L , Hu Z , et al. Dysregulation of immune response in patients with COVID‐19 in Wuhan, China. Clin Infect Dis. 2020;71:762‐768. 32161940 10.1093/cid/ciaa248 PMC7108125

[17] Zhao Q , Meng M , Kumar R , et al. Lymphopenia is associated with severe coronavirus disease 2019 (COVID‐19) infections: a systemic review and meta‐analysis. Int J Infect Dis. 2020;96:131‐135. 32376308 10.1016/j.ijid.2020.04.086 PMC7196544

[18] Li H , Liu L , Zhang D , et al. SARS‐CoV‐2 and viral sepsis: observations and hypotheses. Lancet (London, England). 2020;395(10235):1517‐1520. 10.1016/S0140-6736(20)30920-X PMC7164875 32311318

[19] Zhou Z , Ren L , Zhang L , et al. Heightened Innate Immune Responses in the Respiratory Tract of COVID‐19 Patients. Cell Host Microbe. 2020;27(6):883.e2‐890.e2. 32407669 10.1016/j.chom.2020.04.017 PMC7196896

[20] Yao XH , Li TY , He ZC , et al. A pathological report of three COVID‐19 cases by minimally invasive autopsies. Zhonghua Bing Li Xue Za Zhi. 2020;49:E009. 10.3760/cma.j.cn112151-20200312-00193 32172546

[21] Wang HJ , Du SH , Yue X , Chen CX . Review and prospect of pathological features of corona virus disease. Fa Yi Xue Za Zhi. 2020;36(1):16‐20. 32198986 10.12116/j.issn.1004-5619.2020.01.004

[22] Rea IM , Gibson DS , McGilligan V , McNerlan SE , Alexander HD , Ross OA . Age and age‐related diseases: role of inflammation triggers and cytokines. Front Immunol. 2018;9:586. 29686666 10.3389/fimmu.2018.00586 PMC5900450

[23] Shen YL , Shi YZ , Chen GG , et al. TNF‐alpha induces Drp1‐mediated mitochondrial fragmentation during inflammatory cardiomyocyte injury. Int J Mol Med. 2018;41(4):2317‐2327. 29336470 10.3892/ijmm.2018.3385

[24] Tavener SA , Long EM , Robbins SM , McRae KM , Remmen HV , Kubes P . Immune cell toll‐like receptor 4 is required for cardiac myocyte impairment during endotoxemia. Circ Res. 2004;95(7):700‐707. 15358664 10.1161/01.RES.0000144175.70140.8c

[25] Ackland GL , Abbott TEF , Cain D , et al. Preoperative systemic inflammation and perioperative myocardial injury: prospective observational multicentre cohort study of patients undergoing non‐cardiac surgery. Br J Anaesth. 2019;122(2):180‐187. 30686303 10.1016/j.bja.2018.09.002 PMC6354048

[26] Driggin E , Madhavan MV , Bikdeli B , et al. Cardiovascular considerations for patients, health care workers, and health systems during the COVID‐19 pandemic. J Am Coll Cardiol. 2020;75(18):2352‐2371. 32201335 10.1016/j.jacc.2020.03.031 PMC7198856

[27] de Abajo FJ , Rodriguez‐Martin S , Lerma V , et al. Use of renin‐angiotensin‐aldosterone system inhibitors and risk of COVID‐19 requiring admission to hospital: a case‐population study. Lancet (London, England). 2020;395:1705‐1714. 10.1016/S0140-6736(20)31030-8 PMC7255214 32416785

[28] Rizk JG , Kalantar‐Zadeh K , Mehra MR , Lavie CJ , Rizk Y , Forthal DN . Pharmaco‐Immunomodulatory Therapy in COVID‐19. Drugs. 2020;80(13):1267‐1292. 32696108 10.1007/s40265-020-01367-z PMC7372203

[29] Fernández‐Ruiz M , López‐Medrano F , Asín MAP , et al. Tocilizumab for the treatment of adult patients with severe COVID‐19 pneumonia: a single‐center cohort study. J Med Virol. 2020. 10.1002/jmv.26308 PMC7404673 32672860

